# A role for the membrane protein M6 in the *Drosophila* visual system

**DOI:** 10.1186/1471-2202-13-78

**Published:** 2012-07-04

**Authors:** María Paula Zappia, Guillermo Bernabo, Silvia C Billi, Alberto C Frasch, María Fernanda Ceriani, Marcela Adriana Brocco

**Affiliations:** 1 Instituto de Investigaciones Biotecnológicas “Dr. Rodolfo Ugalde”, Instituto Tecnológico de Chascomús, Consejo Nacional de Investigaciones Científicas y Técnicas, Universidad Nacional de General San Martín, IIB, INTECH, CONICET-UNSAM, 25 de Mayo y Francia, Edificio IIB, B1650KNA, San Martín, Provincia de Buenos Aires, Argentina; 2 Laboratorio de Genética del Comportamiento, Fundación Instituto Leloir and Instituto de Investigaciones Bioquímicas, Buenos Aires (IIB-BA, CONICET), Fundación Instituto Leloir, Av. Patricias Argentinas 435, 1405BWE, CABA, Buenos Aires, Argentina

**Keywords:** Myelin PLP family, Gpm6a, Eye development, Phototactic behavior, Lifespan, Protrusion/filopodium formation, Cell remodeling

## Abstract

**Background:**

Members of the proteolipid protein family, including the four-transmembrane glycoprotein M6a, are involved in neuronal plasticity in mammals. Results from our group previously demonstrated that M6, the only proteolipid protein expressed in *Drosophila*, localizes to the cell membrane in follicle cells. M6 loss triggers female sterility, which suggests a role for M6 in follicular cell remodeling. These results were the basis of the present study, which focused on the function and requirements of M6 in the fly nervous system.

**Results:**

The present study identified two novel, tissue-regulated M6 isoforms with variable N- and C- termini, and showed that *M6* is the functional fly ortholog of *Gpm6a*. In the adult brain, the protein was localized to several neuropils, such as the optic lobe, the central complex, and the mushroom bodies. Interestingly, although reduced M6 levels triggered a mild rough-eye phenotype, hypomorphic M6 mutants exhibited a defective response to light.

**Conclusions:**

Based on its ability to induce filopodium formation we propose that M6 is key in cell remodeling processes underlying visual system function. These results bring further insight into the role of M6/M6a in biological processes involving neuronal plasticity and behavior in flies and mammals.

## Background

Neural plasticity is the mechanism by which information is stored and maintained within individual synapses, neurons, and neuronal circuits to guide organism behavior. Neurite growth and remodeling represents a fundamental process during nervous system development, plasticity, and behavior. Although neuronal plasticity allows the organism to adapt to a constantly evolving environment, little is known about the molecular components and pathways that support it*.* Several myelin proteolipid protein (PLP) family members, such as M6a, M6b, and DM20 [1], have been shown to be involved in neurite outgrowth and filopodium formation [2,3]. In addition, all PLP family members share a common structure, which includes two extracellular loops that potentially interact with external ligands, and four transmembrane domains. The PLP family is widely evolutionarily conserved from arthropods to mammals [4,5]. M6a, a membrane glycoprotein, is prominently expressed in the central nervous system, in particular in the hippocampus, cortex, forebrain, cerebellum, and retina [1,6,7]. Several lines of evidence showed M6a participation in neural development, such as neurite extension and/or filopodium/spine formation in hippocampal [3], retinal [8], and cerebellar [6] neurons, as well as in axonal growth [9]. Indeed, M6Ab, a zebra fish paralog of M6a, also exhibits similar functions [10]. M6a may also be required for filopodium motility and synaptogenesis [8,11,12] and has been implicated in neuronal differentiation of human stem cells [13] and PC12 cells [14].

Chronic social and physical stress decreases *Gpm6a* mRNA levels in the hippocampus, and this downregulation is prevented by administration of antidepressants [15,16], which suggests that M6a participates in plastic hippocampal changes observed in stressed/antidepressant-treated animals. However, the underlying mechanisms remain poorly understood. Interestingly, *M6b* and *DM20* are also regulated by chronic stress [2]. In contrast, *PLP* mRNA, a *DM20* splice variant, which is abundantly expressed in myelin of the central nervous system, is not regulated by stress [2]. However, PLP participates in maintaining structural integrity of the myelin membrane. PLP and DM20 have also been shown to form a complex with integrins in oligodendrocytes [17].

Previous work depicting major steps in PLP evolution identified *M6* as the ancestral gene of the PLP family present in *Drosophila*, maintaining a high degree of conservation in gene structure and amino acid sequence of the predicted protein compared with mouse M6a [5,18,19], suggesting that M6 is the M6a fly ortholog. Therefore, the role of M6a was analyzed in *Drosophila* in the present study. In a previous study, we demonstrated that M6 localizes to the membrane of the ovary follicular epithelium, and M6 knockdown triggers female sterility [20]. Loss of M6 in follicle cells also impairs eggshell formation and epithelial integrity, as well as organization. Therefore, M6 plays an essential role in follicular epithelia maintenance, likely *via* membrane cell remodeling [20]. However, to date, there is no experimental evidence for M6 functional conservation, localization, or function in the fly nervous system.

To address the role of M6a in an intact nervous system, M6 relevance was characterized in adult flies. Results identified novel M6 isoforms that were differentially expressed in the ovaries and heads. All M6 isoforms were structurally and functionally conserved, with one exception; this isoform exhibited a different subcellular localization most likely due to an altered protein structure, thereby giving rise to a non-functional isoform. M6 localization was detected in several brain structures, most remarkably in the optic lobe neuropil. In addition, *M6* mutant flies exhibited a defective response to light. These results identified M6 as one of the molecular components underlying phototactic behavior, and together with M6 localization in the optic lobe, results suggests that M6 might play a role in the fly visual system.

## Methods

### Fly strains

Flies were grown and maintained at 25 °C under a 12 h light/dark (LD) cycle in vials containing standard cornmeal-agar medium. A *w*^*1118*^ stock was used as the control. Potential *M6* mutant stocks *y*^*1*^*w*^*67c23*^; P{EPgy2}M6^EY07032^*w*^*1118*^; P{GT1}M6^BG00390^ and *w*^*1118*^; Mi{ET1}M6^MB02608^/ TM3, Sb^1^ Ser^1^ were obtained from the Bloomington Stock Center [21] and were renamed *M6*^*01*^*M6*^*02*^ and *M6*^*03*^, respectively [20]. The CA06602 stock (*M6*^GFP^) was obtained from the GFP Protein Trap Database at the Carnegie Institution [22]. The *M6*^*01*^*M6*^*02*^ and *M6*^GFP^ strains were backcrossed several generations to *w*^*1118*^ to minimize background effects. The original P-element (EY07032) from *M6*^*01*^ was removed with the transposase (Δ2–3) and the reverted P-excised allele was kept as M6^Δ*01*-rev^ (for details see [20]).

### mRNA isolation, RT-PCR and quantitative real time reverse transcription polymerase chain reaction (RT-qPCR)

Heads and ovaries were dissected from well-fed young flies and homogenized in Trizol Reagent (Life Technologies, Carlsbad California, USA) to isolate total RNA according to manufacturer´s instructions. Then, polyA + mRNA was purified using the PolyATract mRNA Isolation System (Promega, Madison, WI, USA). Complementary DNA was synthesized using oligo dT and SuperScript^TM^ II Reverse Transcriptase (Life Technologies, Carlsbad California, USA).

**Figure 1:**
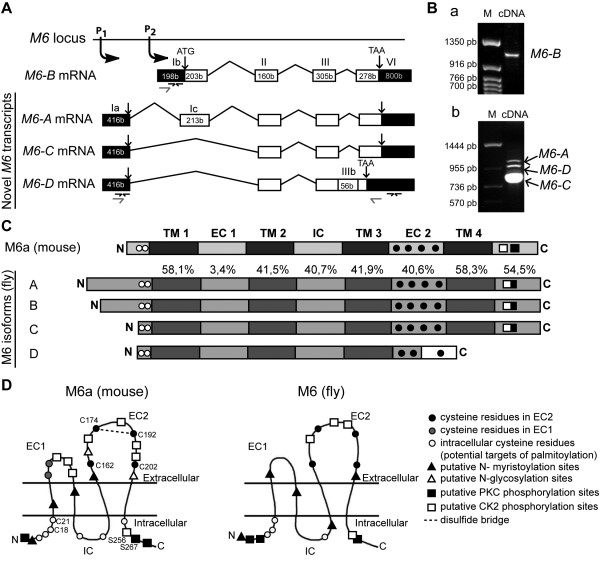
**M6 isoforms are structurally conserved in*Drosophila.*** (**A**) Schematic diagram (not scaled) of the *M6* locus shows the novel *M6-AM6-C* and *M6-D* transcripts from promoter 1 (P1) and *M6-B* from P2. Exons (boxes), introns (lines), untranslated regions (UTR, dark boxes) and coding regions (white boxes) are represented. Numbers in each box indicate nucleotide length (in bases). Roman numbers denote exons according to Schweitzer J. et al [5]. The sequences for *M6-A/C*/*D* are [GenBank: JN872491, JN872492, and JN872493], respectively**.** The previously reported sequences M6-1 [GenBank: AAF71284] and M6-2 [GenBank: AAF71285] correspond to M6-C and M6-B isoforms, respectively. Primers to quantify *M6* levels by RTqPCR are indicated by black arrows. (**B**) Detection of *M6* expression by RT-PCR. cDNAs from *w* heads were amplified using primers (gray arrows) annealing on (**a**) the exon Ib (5’UTR of *M6-B*) and the exon VI (coding region) and on (**b**) the exon Ia (5’UTR of *M6-A/C/D*) and the exon VI (coding region). M, nucleotide marker. (**C**) Schematic representations of M6a and M6 protein topology (not scaled) predicted from the PredictProtein server. Transmembrane domains (TM, dark gray boxes), extracellular and intracellular loops (EC and IC, gray boxes) are flanked by cytoplasmic N and C. Amino acid similarities among each M6-isoform domain are relative to M6a (in percentage). M6-A/B/C are identical between them in each domain except for the N. The frame shift sequence (white box) of M6-D isoform is indicated. Conserved Cys residues C18/21 and C162/174/192/202 in N and EC2 (black and white circles) are according to mouse M6a sequence. (**D**) Putative post-translational modification sites conserved between M6a and M6. These sites were predicted using the PROSITE motif search. The M6 representation corresponds to M6-A/B isoforms. M6-C/D do not have either the putative phosphorylation sites or the N-myristoylation site. M6-D isoform also lacks the CK2 phosphorylation, the N-myristoylation sites from the EC2 domain and the PKC and CK2 phosphorylation sites corresponding to the C. CK2 and PKC phosphorylation sites (white and black squares, respectively) are conserved at the C. N, N-terminus; C, C- terminus.

qPCRs were carried out in a 7500 Real-Time PCR System (Applied Biosystems, Foster City, California, USA). Quantitation of each cDNA was achieved using SYBR Green PCR Master Mix (Applied Biosystems) in triplicate. Primer sequences for housekeeping genes and *M6* 3’UTR were published elsewhere [20]. The oligonucleotide sequences used were: 5’AGAAATTCCAACGCAACTAACAAA3’ and 5’TGTTTCCAACTGGCAATGCA3’, forward and reverse primers, respectively, for *M6-A/C/D* variants (P1, black arrows, Figure1A); 5’TCACTGTGTGCCGTTTAGCTTG3’ and 5’TTTATGGAGTCGAAGTCGGAATTT3’ forward and reverse primers, respectively, for the *M6-B* variant (P2, black arrows, Figure1A). Normalization was accomplished using *Rp49* and *gapdh* as housekeeping genes and resulted in almost identical patterns. Relative quantification was performed using a comparative CT method [23,24]. Before each experiment, the calibration curves were validated. Samples whose curves amplified out of the calibrated dynamic range were eliminated. All procedures followed the manufacturer’s instructions.

### M6 transcripts identification, topology and motif predictions

PCR were done with GoTaq® DNA polymerase according to the manufacturer’s instructions (Promega, Madison, WI, USA). The oligonucleotide sequences used were: 5’AATTCCCAACGCAACTAACAAATTG3’ and 5’CTGTACTCCAGCTCGTTCAGGTT3’, forward and reverse primers for *M6-A/C/D* variants (gray arrows, Figure1A); and 5’ATTCGTTGCTCGGTGGTTATTG3’ and 5’CTAGAAGCGATCCTTCGA3’, forward and reverse primers for *M6-B* (gray arrows, Figure1A). The PCR amplicons were cloned into pGEM®-T Vector System (Promega, Madison WI, USA) and sequenced. cDNA sequences corresponding to novel *M6* variants have been deposited in GenBank [25]. NCBI accession numbers are as follows: [GenBank: JN872491] (*M6-A*), [GenBank: JN872492] (*M6-C*) and [GenBank: JN872493] (*M6-D*). The forward and reverse primers annealing 38b downstream of the P1 site and 367b downstream of the stop codon were: 5’TTTTGAGCGAATTCAGTTGG3’ and 5’GCATTCGGCAATTCAGAAGAA3’, respectively.

Bioinformatic analysis included the Vector NTI Advance 10 software package, Kalign (2.0) and ClustalW (1.83) alignments [26]; the Ensembl website was used to determine exon/intron boundaries [27]. In addition, Predict Protein Server [28,29], which includes PHD predictions for protein topology, PROF predictions for motif scan (PROSITE) and DISULFIND for disulfide bridge prediction, were employed.

### Cloning and plasmids used

To obtain GFP::M6-A, GFP::M6-B, GFP::M6-C and GFP::M6-D plasmids, all cDNAs were cloned into pGEM®-T Vector System (Promega) and subcloned into pEGFP-C1 vector (BD Biosciences Clontech) with Pfu DNA polymerase (Promega) according to the manufacturer’s recommendations. Primers were designed to amplify the complete coding sequences (CDS) from the initial ATG to the stop codon. Primers included the SacI and KpnI restriction recognition sites to allow in-frame cloning into the pEGFP-C1 multiple cloning sites. Oligonucleotide sequences used were: 5’GAGCTCAAATGGCGTTGTGAAGTATG3’, 5’GAGCTCAAATGCCGGGCAAGGGGAACAA3’ and 5’GAGCTCAAATGGGAGAATGCTGCCAAT3’, forward primers for *M6-AM6-B* and *M6-C/D*, respectively; 5’GGTACCCTAGAAGCGATCCTTCGAGGT3’ and 5’GGTACCTCATGTCCTCCAGTTTCGTGTT3’, reverse primers for *M6-A/B/C* and *M6-D*, respectively. After the cloning step, plasmids were sequenced to exclude mutations. Control vectors, GFP::M6a [12] expressed in the plasma membrane and GFP (GFP-PH, plasmid 21179, Addgene, Cambridge, MA, USA, [30]), which bind to plasma membrane phospholipds, were found to be enriched at the plasma membrane.

### Cell line and transfections

Mouse neuroblastoma 2a (N2a, Clone CCL-131 ATCC, Manassas, VA, USA) cells were cultured in DMEM with 10-20 % (v/v) fetal bovine serum, penicillin, and streptomycin.

For transfections, we used polyethylenimine (PEI, School of Pharmacy and Biochemistry, UBA, Buenos Aires, Argentina). Briefly, 2 μg of plasmid DNA and 3 μl of 25 mM PEI were diluted in 50 μl of protein and antibiotic free medium (OPTI-MEM I Reduced Serum Medium, Gibco) and incubated for 8 minutes. Next, 200 μl of complete medium were combined with transfection mix and added to each well in a 24 wells per plate format, containing cells previously washed twice with PBS (phosphate buffered saline). Cells were incubated with the transfection mix for 2 hours at 37 °C. Then, cells were washed 3 times with PBS and incubated with complete fresh medium.

### Cell staining and image analysis

Twenty-four hours after transfection, N2a cells were fixed in 4 % paraformaldehyde/4 % sucrose in PBS for 15 min at 4 °C. For F-actin staining, permeabilization was carried out and stained with 0.1 % Triton X-100 in PBS for 2 min. Cultures were blocked with 3 % BSA in PBS for 1 hour, followed by incubation with the rhodamine phalloidin 1/1,000 (Molecular Probes, Eugene, OR) in 3 % BSA in PBS at 37 °C for 1 hr. Coverslips were incubated with DAPI and then were mounted with FluorSave Reagent (Calbiochem, La Jolla, CA). Fluorescent images were acquired by using a Nikon Eclipse 80i microscope (60x/1.4 objective) equipped with CoolLED pE excitation system or a confocal laser scanning microscope (Zeiss LSM510 Meta, 63x/1.4 objective).

The percentage of cells displaying filopodial protrusions (visualized by the F-actin marker phalloidin) was calculated for both transfected as well as non-transfected cells from the same coverslip. The percentage of non-transfected cells bearing filopodia from the same coverslip was used to normalize data. The ratio of transfected to non-transfected filopodium-bearing cells from the same coverslip was determined. A ratio similar to one implies that filopodium formation was not induced. At least 80 cells per coverslip were analyzed for 4 replicates from each experiment. Each experiment was independently repeated three times. Images were processed using Photoshop and Illustrator (Adobe Systems).

### Whole brain immunohistochemistry and image analysis

Adult brains from 3–6 day-old flies were dissected, fixed and stained as previously described [31]. Briefly, heads were fixed in 4 % paraformaldehyde in PB (100 mM KH2PO4/Na2HPO4) for 30 minutes to 1 hour at room temperature. The brain was dissected and washed with 0.3 % Triton X-100 in PBS (PT). Brains were then blocked in 7 % goat serum in PT for 1 h at room temperature and incubated with the primary antibody in PT (0.6 % Triton X-100) for 48 h at 4 °C. Washes were carried out in PT (0.6 % Triton X-100) for 20 minutes and repeated twice prior to the addition of the secondary antibody. After a 2 h incubation step, brains were washed for three times in PT (0.6 % Triton X-100), once in PT (0.1 % Triton X-100) and mounted in FluorSave Reagent (Calbiochem). All steps were carried out at room temperature unless otherwise indicated. The primary antibodies used were mouse anti-FasII (1D4, 1/5, Developmental Studies Hybridoma Bank [DSHB], IA, USA), anti-elav (9F8A9, 1/10, DSHB), and rabbit anti-GFP (1/300, Molecular Probes, Invitrogen Carlsbad, CA USA). Secondary antibodies conjugated to Cy2 or Cy3 were used (1/500, Jackson ImmunoResearch Laboratories West Grove, PA, USA). Detection of GFP::M6 in the adult brain was repeated at least three times examining 8–10 brains in each experiment. Brains from *white* flies were used as a negative control to confirm the specificity of the GFP antibody. Fluorescent images were acquired with the laser scanning confocal microscope Zeiss LSM510 Meta using 20x/0.8, 40x/1.3 and 63x/1.4 objectives. Images were processed using Photoshop and Illustrator (Adobe Systems).

### Lifespan analysis

Survival was determined at 25 °C under LD conditions. One hundred male flies from each genotype were maintained in vials (10 flies/vial) containing standard medium. Flies (0–48 h old) were placed in vials and were scored for survivorship every 3–4 days, when they were transferred to fresh vials to minimize death caused by bacterial infection or moist in the medium. Three independent experiments were carried out. Survival curves represent the percentage of surviving flies as a function of time. For statistical analysis the mean life span of each strain was calculated as the time (in days) at which survival reached 50 % of the starting population. In all experiments, only males were used because female life span is known to depend upon reproductive history [32].

### Environmental scanning electron microscopy (ESEM) of adult compound eyes

Young adult male flies (3–6 days) were collected, anesthetized and immobilized on the ESEM mount using water-based colloidal carbon glue for proper orientation. The electroscan was performed with an environmental scanning electron microscope (ESEM, model XL30, Philips) at 20.0 kV and 0.9 Torr in the auxiliary mode. This technology does not require metal coating of the specimen.

### Phototactic behavior

Before each assay, 40 adult males of 2- to 3-days old were selected under CO_2_ and allowed to recover in fresh food vials for 1–3 days in LD. Phototactic behavior was performed as previously described [31]. Briefly, a horizontal device allows the “collecting tubes” to slide through the one containing the flies at the beginning of the experiment, which is always kept in the same position in reference to the light source. At least 15 min before testing flies were transferred to darkness for adaptation; further manipulations were performed under a safe red light. Flies were moved to a “test” tube (13 cm long, 1 cm wide). Five “collecting” tubes were placed opposite to the test one. The white cold light source (150 Watt quartz halogen fiber optic illuminator, Fiber-Lite MI-150) was initially placed right behind the collecting tube 1, and kept in line with the test tube throughout the experiment. Each collecting tube was allowed to connect sequentially with the test tube for 1 min. Thus, flies were allowed to freely move to the collecting illuminated tube for 1 min, and then the tube was moved to the next position. The number of flies in each (collecting and test) tube was counted, and the proportion of flies that had a positive phototactic response (defined as those that moved towards the light within the first 2 min of initiating the test, or stayed in the first 2 collecting tubes) was analyzed. In each experiment, the results were the mean of the scores from 2 trials recorded from 40 flies per genotype. Each experiment was independently repeated 5 times.

### Total fly locomotor activity measurement

Spontaneous fly locomotor activity of 2-3-days old adult males was monitored by recording infrared beam crossings in glass tubes (6.5-cm length, 3-mm inside diameter) using a commercially available *Drosophila* activity monitoring system (TriKinetics, Waltham, MA). Individual activity was scored under LD conditions for 3 consecutive days. Total activity levels were determined as total counts per day displayed for each fly. Statistical analysis included a Kruskall-Wallis test. Data were obtained from at least three independent experiments; n = 30 flies per genotype in each experiment.

### Statistical data analysis

Graphs were generated with GraphPad Prism software. Statistical analysis was performed with IS (Infostat software, Grupo InfoStat, FCA, Universidad Nacional de Córdoba, Argentina). Group means were analyzed for overall statistical significance by one-way analysis of variance (ANOVA) followed by multiple comparison tests. Non-parametric analysis was performed (Kruskal-Wallis followed by multiple comparison test) when assumptions on the normal distribution and variance did not allow otherwise.

## Results

### Novel M6 variants are generated by alternative splicing

The *M6* gene is located in 78D4 of chromosome 3 L. The complete *M6* exon-intron structure comprises 4–5 exons, which span a genomic interval of 4.9 kb. Two depicted transcription initiation sites (P1 and P2) give rise to four *M6* mRNA variants (Figure1A) and four predicted proteins that contain 187 to 319 amino acids (aa).

Early in the study, only isoforms *M6-A* (CG7540-RA, [GenBank: NM141066]) and *M6-B* (CG7540-RB, [GenBank: NM168911]) had been reported as transcript variants encoded by the *M6* gene in the FlyBase [33], with both transcripts producing apparently the same protein, M6 A-B (314 aa). RT-PCR analysis of wild-type ovaries and heads with primers annealing to the end of isoforms *M6-A* or *M6-B* (gray arrows, Figure1A) resulted in the identification of two novel shorter *M6* transcripts (~0.8 kb, Figure 1Bb), as well as the expected *M6* transcripts (~1 kb). Sequence analysis indicated that shorter cDNAs were novel variants expressed in *D. melanogaster*. The novel *M6* transcripts, termed *M6-C* [GenBank: JN872492] and *M6-D* [GenBank: JN872493], are two variants derived from alternative splicing. Both novel transcripts initiate at P1 and lack the first coding exon (exon Ic), thereby giving rise to an N-terminus shorter than the one in M6-A (Figure1A-C). The M6-C predicted protein has 248 aa. Previous work reported sequences for *M6* in *Drosophila* ([GenBank: AF253528]; [5,18,19]) as M6-1 [GenBank: AAF71284] and M6-2 [GenBank: AAF71285]. Through sequence alignment, we determined that those sequences correspond to M6-C and M6-B isoforms, respectively, described in the present study (data not shown). In addition, *M6-D* has 56 additional bases (b) resulting from retention of the last intron, which shifts the reading frame and creates a premature stop codon located 183 bases upstream of the *M6-A* stop codon (exon IIIb). Therefore, this *M6-D* variant is predicted to give rise to a shorter protein (187 aa, Figure1C), with 31 novel aa at the C-terminus (Figure1 A-C). In addition, there was a difference between the *M6-A* variant reported in the database (CG7540-RA; [GenBank: NM141066]) and the cloned variant from the present study [GenBank: JN872491]. The new variant has 15 additional base pairs, 10 of which are within the first coding exon (exon Ic). Therefore, the predicted protein has 319 aa, with an N-terminus slightly longer than M6-A reported in the database. Additional primers were used to confirm these results (see Figure1 for details), suggesting that the novel variants (*M6-C* and -*D*) contain the 5’UTR corresponding to the P1 promoter and share the same 3’UTR (data not shown).

### M6 is structurally conserved

Using the ‘Predict Protein’ Server [29] and other bioinformatic tools, it was possible to predict the M6 tertiary structure. M6, mammalian M6a, and other PLP family members comprise four transmembrane domains (TM), a minor (EC1) and a major (EC2) extracellular loop, one intracellular loop (IC), and both N- and C- termini, which were localized within the cytoplasm. Figure1C shows the comparison at the primary structure level between M6a (mouse) and M6 (fly) isoforms. The similarity percentage between different domains of M6a and M6 is also noted. The TM domains, EC2, IC, and C-terminal regions share a high similarity (40-60 %) between mouse and fly sequences.

In addition, alignment of all M6 isoforms revealed that M6-A, -B, and -C are identical, with exception of the N-terminal region. M6-A exhibits a longer N-terminus than M6-B, whereas M6-C and -D display a shorter one. Variability at the N-terminal region is due to alternative initiation sites (P1 and P2), as well as alternative splicing of the first coding exon (exon Ic, Figure1A). In contrast, M6-D lacks the last transmembrane domain. Therefore, the C-terminus is localized outside the cell, which suggests that the major extracellular loop (EC2) does not adopt a proper conformation owing to the frame shift and premature stop codon.

We also sought for putative target sites for posttranslational modifications within the M6 sequence. The analysis revealed several conserved motives for phosphorylation (casein kinase 2 (CK2) and phosphokinase C (PKC)), N-myristoylation, and key cysteine (Cys) residues. Interestingly, these sites are conserved in mammalian M6a. Predictive models of tertiary structures with sites conserved between mouse M6a and fly M6 are shown in Figure1D. EC2 Cys residues forming disulfide bonds essential for M6a function [12], and two N-terminal Cys residues, which are conserved in all PLP family members of vertebrate and invertebrate organisms [5], are present in most M6 isoforms (black and white circles, respectively, Figure1C-D). In particular, fly M6 and mouse M6a share three Cys residues at the intracellular N-terminus. In contrast, the M6-D isoform lacks the third Cys residue in EC2, and a fourth Cys residue appears in the frameshifted sequence. CK2 and PKC phosphorylation sites at the C-terminus (black and white squares, respectively, Figure1 C-D) correspond with M6a phosphorylated S256 and S267 sites [34,35], which could be crucial for M6a function [10,11,14].

### M6 is functionally conserved

**Figure 2:**
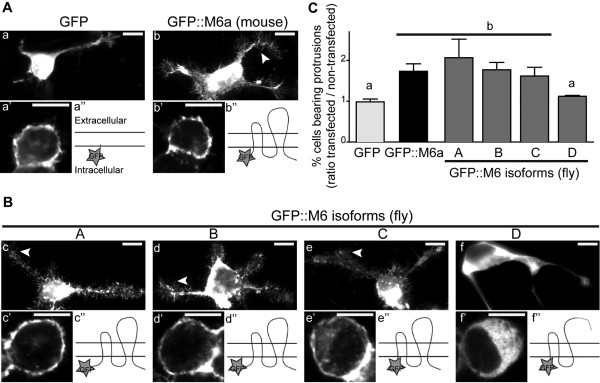
**M6 isoforms are functionally conserved in*Drosophila.*** Twenty-four hours after transfection N2a cells were fixed and stained with phalloidin to visualize F-actin and assess filopodium formation. (**A-B**) Fluorescent images of transfected N2a overexpressing GFP (a), GFP::M6a (b) and the fruit fly GFP::M6 isoforms (c-f). Arrowheads indicate filopodial protrusions. Confocal images showing the subcellular localization of GFP (**a’**), GFP::M6a (**b’**) and the GFP::M6-A, -B and -C isoforms (**c’, d’, e’**) at the cell surface. In contrast, the M6-D isoform (**f’**) mostly localized to the intracellular compartment. Scale bar is 10 μm. A schematic representation of the protein conformation (**a”, b”, c”, d”, e”, f”**) is also included. (**C**) M6 overexpression induced filopodium formation in N2a cells**.** The ratio of transfected to non-transfected filopodium-bearing cells from the same coverslip were determined. A ratio value similar to one implies that filopodium formation was not induced. Mean ± SEM; N = 3. Statistical analysis included a reciprocal data transformation (multiplicative inverse) and a Randomized Block ANOVA (p < 0.05) followed by a BBS multiple comparison. Different letters indicate significant differences.

Because M6 isoforms are predicted to be transmembrane proteins (Figure1C-D and 2 Bc”, d”, e”, f”) and M6a is localized to the plasma membrane [12], localization of the various M6 variants was analyzed. M6 isoforms tagged to GFP were overexpressed in murine neuroblastoma 2a (N2a) cells, and localization was determined by confocal section analysis. Similar to mammalian M6a (Figure2A b’), M6-A, -B, and -C exhibited cell surface expression (Figure2B c’-e’). In contrast, M6-D, which lacks the fourth TM and EC2 domains, was mostly restricted to intracellular compartments (Figure 2Bf’).

Mouse M6a has been shown to induce filopodium/spine formation in neural and non-neural cells [3]. Therefore, to determine functional conservation of the distinct fly M6 variants, filopodium formation was analyzed. Briefly, N2a mammalian cells were transfected with fly M6 isoforms or mouse GFP-M6a, as well as GFP alone as the control (Figure 2Aa-b, Bc-f, 2 C). The percentage of cells bearing filopodial protrusions was quantified for transfected, as well as non-transfected cells through visualization of the F-actin marker phalloidin. The ratio of transfected to non-transfected filopodium-bearing cells was calculated to measure variant-specific overexpression resulting in filopodium formation. GFP overexpression did not induce filopodium formation (ratio = 1), whereas GFP::M6a overexpression exhibited significant induction (approximately 2-fold, Figure2C). Overexpression of M6-A, -B and -C isoforms induced filopodium formation similar to mammalian M6a, which significantly varied from GFP alone (*P* < 0.05, Figure2C). In contrast, M6-D, the truncated isoform lacking the major extracellular loop (EC2) and the fourth TM domain (Figure 2Bf”), did not induce filopodium formation (Figure2C). These results suggest that M6-A, -B, and -C are functionally conserved variants. In addition, altered protein structure was likely responsible for M6-D subcellular delocalization and subsequent functional loss. These results suggest that the novel M6-D variant encodes a non-functional isoform.

### M6 isoforms are differentially expressed

**Figure 3:**
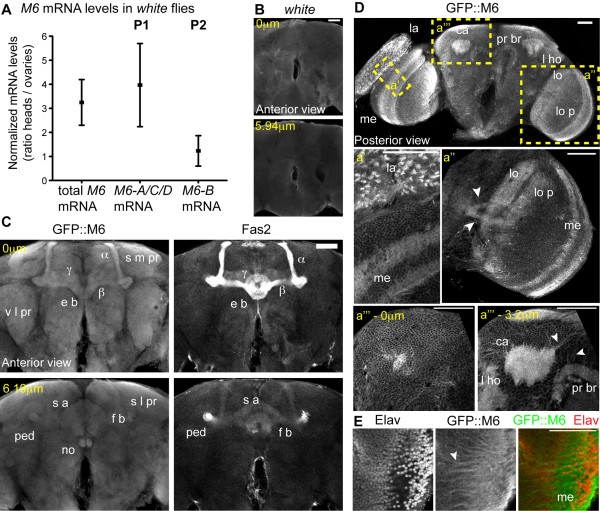
**M6 localizes to neuropils and projections in the adult brain.** (**A**) *M6* levels measured in control (*w*) ovaries and heads by RT-qPCR. Total *M6* mRNA was quantified using primers directed to the 3’UTR of all *M6* isoforms. Levels of *M6-A*/*C/D*, derived from promoter 1 (P1, left panel) and *M6-B*, from P2 (right panel), were quantified using primers annealing at the 5’UTR of the P1- or the P2-transcripts (exon Ia and Ib, respectively; black arrows in Figure1A). The ratios between normalized *M6* expressions in heads relative to ovaries are plotted. Ratio of mean ± SEM, n = 3. **(B-E)** M6 localization was analyzed using a GFP protein trap (*M6*^GFP^) that expresses endogenous levels of GFP-tagged M6 isoforms [20]. Brain immunofluorescence of control *white* (**B**) or *M6*^GFP^ (**CE**) adults stained with GFP, FasII (neuropil marker, **C**) and Elav (neuronal marker, **E**). Magnified views of the regions indicated in **D** are in **a’a”** and **a”’**. Single confocal sections of brain frontal views are shown (anterior view in **C** (0 μm); posterior view in **D**). Two depths of a Z-stack from the same brain are presented in **B** (0 μm and 5,94 μm), in **C** (0 and 6.19 μm), and in **D a”’** (0 and 3.2 μm). The neuropils labeled in *M6*^GFP^ included: lamina (la), outer and inner medulla (me), lobula (lo) and lobula plate (lo p), calyx neuropil (ca), pedunculus (ped), Kenyon cells (α, β and γ), ellipsoid body (e b), superior arch (s a), fan shaped body (f s), noduli (no), protocerebral bridge (pr br), lateral horn (l ho), superior medial protrocerebrum (s m pr), ventrolateral protocerebrum (v l pr) and superior lateral protocerebrum (s l pr). Cortical neuronal cell body layer at the surface of the brain is shown (**D a”’**-0 μm). (**E**) Magnified views of the medulla. White arrowheads point to projections (**ED a”, a”’** (3.2 μm)). Scale bar, 50 μm.

Because *M6a* is highly expressed in the mammalian nervous system, *M6* mRNA expression was analyzed in *Drosophila* heads. Using qPCR, we observed prominent expression in the heads compared with *M6* ovary expression (Figure3A). Subsequently, we specifically quantified mRNAs produced by either P1 or P2 promoters in wild-type heads and ovaries (Figure3A) using primers that annealed to the corresponding 5’UTR (black arrows, Figure1A). The *M6-B* transcript from P2 was expressed at similar levels in both samples. However, a predominance of P1-derived transcripts (*M6-A, -C* and *-D*) was detected in the heads. These results reveal tissue-specificity in the *M6* promoters, where P1-derived transcripts are most abundantly expressed in fly heads.

### M6 localizes to several brain structures and neural projections

Antibodies specific to M6 were generated to determine whether M6 protein was expressed in the *D. melanogaster* nervous system. However, the results were inconclusive. Therefore, we used the fly line *M6*^GFP^, which reports endogenous levels of M6. Our previous results demonstrated that *M6* mRNA levels in *M6*^GFP^ are not reduced compared with control flies, and only P1-specific isoforms were tagged in frame to GFP at the N-terminus [20]. Immunofluorescence images of whole-mount brains of young adult flies are shown in Figure3B-E. Distinct neuropils were identified with antibodies specific to FasII (Figure3C). In addition, antibodies specific to Elav, a neuronal-specific marker, served as the counterstain (Figure3E). In *M6*^GFP^, a general staining of the major brain centers at the protocerebrum (comprising optic lobe, mushroom bodies, and central complex, Figure3C-D) was observed. In fact, GFP::M6 was expressed in most mushroom body structures (calyx neuropil, pedunculus, and Kenyon cells). In addition, GFP::M6 was expressed in the central body complex, more precisely in the ellipsoid body, superior arch, fan-shaped body, noduli, and the protocerebral bridge (Figure3C). Other regions of the protocerebrum, such as the lateral horn, superior medial protocerebrum, ventrolateral protocerebrum, and superior lateral protocerebrum exhibited M6 expression (Figure3C, D a”’-3.2 μm).

In addition, neuropils most predominantly expressing GFP were present in the visual system (lamina, outer and inner medulla, lobula, and lobula plate) (Figure3D). Interestingly, although entire neuropils were labeled, specific neural projections within the optic lobe that projected to the central brain, medulla, calyx, and protocerebral bridge were also labeled (arrowheads in Figure3D a’ and a”’-3.2 μm, 3E). GFP::M6 localization in several neuropils and in the cortical neuronal cell layer at the brain surface (Figure3D a”’-0 μm), as well as Elav localization, further supported M6 neuronal expression.

### M6 downregulation reduces lifespan

**Figure 4:**
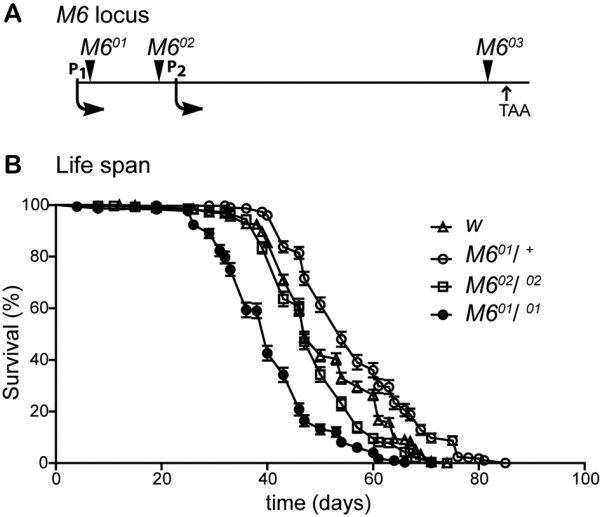
**Median lifespan is reduced in*M6*mutant flies.** (**A**) Schematic diagram (not drawn to scale) of the *M6* locus showing the localization of independent P-element insertions (arrowheads). Start transcription sites corresponding to P1 and P2 and the stop codon (TAA) are also indicated. (**B**) Survival curves of adult *w*, *M6*^*02*^/ ^*02*^, heterozygous and homozygous *M6*^*01*^ males (*M6*^*01*^/ ^+^ and *M6*^*01*^/ ^*01*^, respectively). The percentage of surviving flies ± SE over time (in days) at 25 °C in LD (standard conditions) is shown. Statistical analysis included a Log-rank (Mantel-Cox) survival curve comparison (p < 0.0001; Graphpad Software). Sample size ranged between 292 and 496 flies per genotype.

To determine the role of M6 in *Drosophila* lifespan, potential *M6* mutants containing inserted transposons within the *M6* locus (Figure4A) were characterized. *M6*^*01*^ flies contain a P-element inserted into the first exon (exon Ia), which corresponds to the 5’UTR of *M6-A-C,* and *-D* transcripts, while *M6*^*02*^ contains a different P-element within the first intron. Our group previously demonstrated that *M6*^*01*^/^*01*^ flies are hypomorphic mutants [20]. In the present study, the role of M6 in fly survival was evaluated under normal conditions. The lifespan of *M6*^*01*^ hypomorphic mutants was compared with that of homozygous *M6*^*02*^, heterozygous *M6*^*01*^*,* and *w* (control) flies. The male flies did not exhibit any change in maximal lifespan (85 days for *M6*^*01*^/^*+*^, 71 for *M6*^*01*^/^*01*^*,* and 74 for *M6*^*02*^/^*02*^*vs*. 74 days for *w*, Figure4B). However, when we examined the median lifespan, a parameter that indicates the time in which half of the population has died, significant differences were observed. While *M6*^*02*^/^*02*^ flies behaved similarly to control flies, a significantly reduced median lifespan was detected in homozygous *M6*^*01*^ males compared with both controls (40 ± 3 *vs*. 47 ± 2 and 54 ± 3 days for *M6*^*01*^*/*^*01*^*, w* and *M6*^*01*^/^+^ flies, respectively; *P* < 0.0001). Because the *M6*^*01*^ flies were backcrossed 10 times to *w* flies to homogenize the genetic background, we conclude that the observed reduction in lifespan is indeed due to reduced *M6* expression.

**Figure 5:**
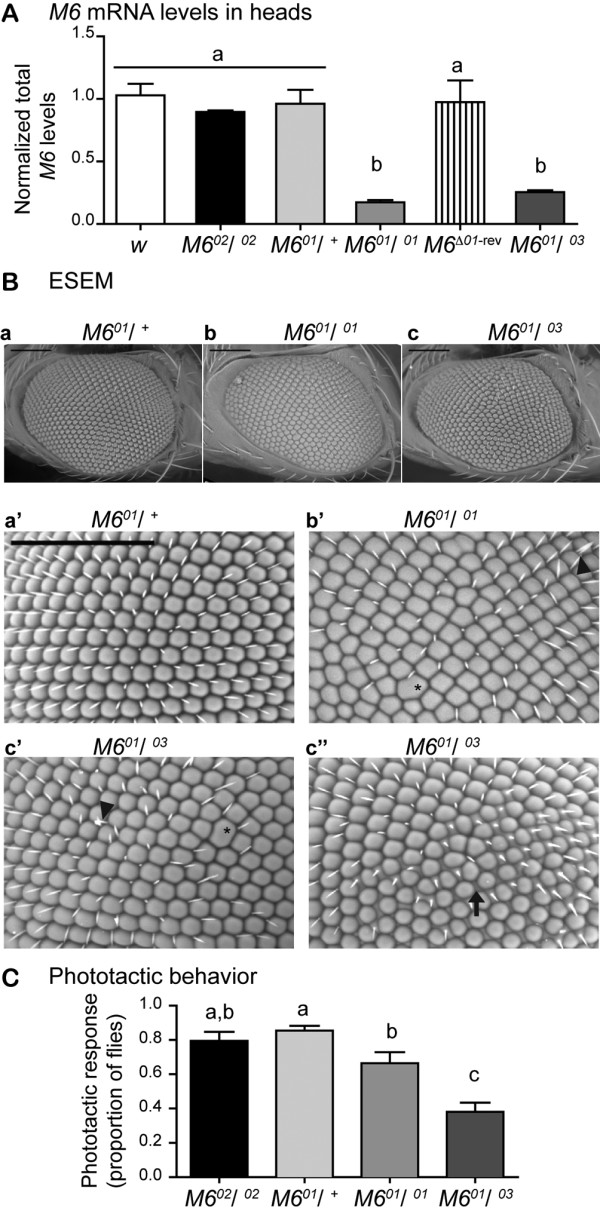
***M6*downregulation triggers a mild rough eye phenotype and impairs the phototactic response.** (**A**) *M6* mRNA levels in heads of control (*w*), *M6*^*02*^/ ^*02*^, heterozygous and homozygous *M6*^*01*^, *M6*^Δ*01*-rev^ (*M6*^*01*^ P-excised), and the trans-heterozygous *M6*^*01*^/ ^*03*^ mutants were assessed by RT-qPCR. The values shown are relative to the control genotype (white bar). Mean ± SEM, n = 3–5. Kruskal-Wallis test, p < 0.001, followed by a multiple comparison test. Different letters indicate significant differences. **(B)** Representative images of young male fly eyes taken with an environmental scanning electron microscope (ESEM). Eyes of heterozygous *M6*^*01*^/ ^+^ used as control (**a**, **a’**), homozygous *M6*^*01*^/ ^*01*^ (**b**, **b’**) and trans-heterozygous *M6*^*01*^/ ^*03*^ (**c**, **c’** and **c”**) are shown**.** Lower and higher magnifications are displayed in the upper and lower panels, respectively. In addition to the disorganized ommatidium array, alterations in the ommatidial shape or fusions between contiguous ommatidia (asterisks) and bristle defects (arrowheads) are indicated. Altered interommadial space was detected in around 20 % of *M6*^*01*^/ ^*03*^ mutants (arrow, **c”**). The ventral-dorsal axis is oriented left to right; scale bar is 100 μm. **(C)** Young male flies were assessed for their response to light in the phototaxis behavioral paradigm. Mean ± SEM, n = 5 independent experiments. Statistical analysis included a two way ANOVA (p < 0.0001) followed by a Newman-Keuls multiple comparison test between genotypes.

This finding opens the provocative possibility that M6 might play a role in regulating animal aging (Figures 4B, 5A).

### Reduced M6 levels result in mild defects in the adult eye structure

*M6* expression was measured in fly heads of control and *M6* mutants (Figure5A). In addition to lines previously described, a transheterozygous *M6*^*01*^/^*03*^ line was included in the analysis. The *M6*^*03*^ line has a P-element insertion in the last coding exon (exon VI) of all functional variants and was determined to be homozygous lethal at the embryonic stage (Figure4A). Heterozygous *M6*^*01*^ and *M6*^*02*^*/*^*02*^ flies exhibited M6 mRNA levels similar to the w control. In contrast, homozygous M6^01^ and transheterozygous *M6*^*01*^*/*^*03*^ flies exhibited significantly reduced M6 mRNA levels compared with control flies (P < 0.01, Figure5A). In addition, in the P-excised *M6*^*Δ01-rev*^ flies, M6 was restored to normal levels, suggesting that the P-element insertion in *M6*^*01*^ reduced M6 expression in the heads.

Characterization of the M6 mutants revealed morphological alterations in homozygous M6^01^ eyes, which suggested that M6 might play a role in this structure. To evaluate M6 participation in eye development, adult eyes were examined by environmental scanning electron microscopy (ESEM). The control flies, w and heterozygous M6^01^, exhibited a stereotypical, uniform, ommatidium pattern (Figure5Ba, and data not shown). In contrast, in the M6 hypomorphic mutants (*M6*^*01*^*/*^*01*^ and *M6*^*01*^*/*^*03*^), a mild rough-eye phenotype was observed, as well as a disorganized ommatidium array, defects in ommatidium shape and/or ommatidium fusion (asterisks in Figure 5Bb’ and c’), and defective or missing bristles (arrowheads). In addition, approximately 20 % of *M6*^*01*^*/*^*03*^ transheterozygote eyes exhibited an excessive interommatidial space (arrow in Figure 5B c”). These results suggest that M6 plays a role during eye development (see below).

### M6 is required for light response

M6 is prominently expressed in the optic lobe, which suggests that M6 might play a role in the visual system, in particular in response to light. Therefore, a phototaxis paradigm was used to detect and process light information. Wild-type adult flies exhibit positive phototactic behavior. Because w flies do not have pigmented eyes, the *M6*^*02*^*/*^*02*^ and *M6*^*01*^*/*^*+*^ flies were used as controls to account for the potential contribution of eye pigmentation. Although there were subtle differences between *M6*^*01*^*/*^*01*^ phototactic responses, the *M6*^*01*^*/*^*01*^ and *M6*^*01*^*/*^*03*^ young males exhibited a significantly defective response compared with controls (P < 0.0001, Figure5C), thereby suggesting that M6 is required for normal responses to light. Although M6 mRNA levels were similarly reduced in *M6*^*01*^*/*^*01*^ and *M6*^*01*^*/*^*03*^ mutants, the phenotypic consequences were greater in *M6*^*01*^*/*^*03*^. Although the exact nature of this difference remains to be determined, it is worth mentioning that the P-element in M6^01^ only affects a subset of splice variants (M6-A/C/D, unpublished data), while the P-element in *M6*^*03*^ is inserted in a region common to all functional variants (M6-A/C and M6-B), likely affecting all isoforms. Our results suggest that the differences in phototactic response could be a result of differential M6 variant expression in each specific mutant.

To rule out that the defective response to light induced by M6 downregulation was due to a general decrease in locomotor activity, adult M6 mutant flies were analyzed in Drosophila activity monitors (Trikinetics, Walthman, MA). Interestingly, *M6*^*01*^*/*^*01*^ and *M6*^*01*^*/*^*03*^ males did not exhibit significant differences compared with control flies (w, *M6*^*02*^, or *M6*^*01*^/^+^; *P* > 0.05, n = 3 independent experiments, data not shown). Therefore, the impaired light responsiveness was more likely derived from higher-order visual processing defects triggered by M6 downregulation.

## Discussion

Results from the present study demonstrate that *Drosophila M6* is expressed in the fly nervous system. In addition, we identified two novel isoforms and further described the molecular organization of the *M6* gene. The expression of most isoforms in neuroblastoma cells resulted in protrusions similar to those previously reported for mouse M6a, which suggests that M6 constitute the functional ortholog of mammalian M6a. In addition, analyses of insertional mutants demonstrated that *M6* is required for a proper adult phototactic response.

### Structural conservation of PLP family members

The generation of transcript variants by distinct promoters and alternative splicing seems to be a common feature in PLP family member genes conserved through evolution. Similar to *Drosophila M6* organization, zebrafish DMβ2 and rat *gpm6a* (isoforms Ia and Ib) exhibit 5’UTR alternative splicing that produces two N-termini [5,36]. The human *GPM6A* gene exhibits three transcripts [GenBank: NM005277, NM201591, NM201592] that differ at the N-terminus owing to specific transcriptional start sites and/or alternative splicing in the first coding exon. Similar features have been described for other members of the PLP family, such as *m6b* and *plp-1*[19]. In the present molecular analysis of *Drosophila M6*, we have identified two novel M6 isoforms with a short N-terminus (M6-C and -D), as well as a third isoform (M6-A) with a longer N-terminus compared with those reported in the database. The M6 isoform variation at the N-terminus is also due to alternative transcription start sites and alternative splicing of the first coding exon (exon Ic). Since M6 is the only PLP family member in *Drosophila*, we propose that the splice variants may tailor M6 according to specific needs of each tissue. In the case of *Drosophila*, we demonstrated that isoform expression is tissue-regulated by differential promoter activity (P1 in heads *vs*. ovaries, Figure3). Similarly, Cooper *et al*. (2009) demonstrated tissue-regulated *gpm6a* expression in the rat; Ia and Ib variants from alternative promoters are differentially expressed in the brain and kidney.

### Functional conservation of PLP family members

Results from the present study demonstrate that M6-A, -B, and -C proteins localize to the cell surface in neuroblastoma cells. As previously shown for mouse M6a [3], all of them induced filopodium formation, demonstrating that *M6* and *M6a* are indeed functional homologs. Interestingly, we also detected a non-functional isoform (M6-D) that was mostly restricted to the cytoplasm. M6-D lacks the third Cys residue from the EC2 domain, as well as the fourth TM domain. Previous work from our group on the mouse M6a shows that the first and fourth Cys residues (C162 and C202) are crucial for cell surface expression and for M6a function [12]. In the case of fly M6-D, the absence of the last TM may prevent proper EC2 folding, resulting in a defective interaction between the first and fourth Cys, thus leading to altered subcellular localization and impaired filopodium formation. Consistent with Fuchsova *et al*. (2009), the EC2 domain structure appeared to be necessary for proper protein localization and function. In addition, PLP mutants lacking Cys residues from EC2 are misfolded and, therefore, are retained in the endoplasmic reticulum [27]. Interestingly, because M6-D is endogenously expressed in ovaries and heads (Figure1A), it is possible that it might act as an endogenous regulator. Intriguingly, *M6* expression is regulated by nonsense-mediated mRNA decay (NMD), which is a post-transcriptional regulation mechanism that targets transcripts containing early stop codons [37]. The *M6-A* transcript is an NMD-target, whereas *M6*-B is not [37]. Because *M6-A* and *M6-D* transcripts share the 5’UTR sequence, it is possible that *M6-D* also undergoes degradation *via* the NMD pathway.

### M6 functions in the visual system

M6 was predominantly expressed in the fly adult optic lobe (Figure3). In the mouse, M6a is present in neuronal processes of the retina, including axons of retinal ganglion cells during development, and inner and outer plexiform layers of adult retina. In addition, M6a overexpression in retinal cells enhances neurite outgrowth *in vitro*[8]. In *Xenopus* and zebrafish, M6a is expressed in the retina, in particular in the inner nuclear layer and ganglion cell layer [5,38]. Considering that the vertebrate and fly visual system share structural and functional molecular mechanisms, as well as developmental features [39], we conclude that M6a/M6/DMb localization in the visual system is conserved through evolution.

Despite previous reports of retinal M6a expression [5,8,38], *in vivo* M6a function in the visual system has not been described. The present study provides the first experimental evidence for the requirement of M6 in the adult response to light (Figure5C). Interestingly, although *M6* hypomorphic mutations resulted in a subtly altered eye structure in the adult (Figure5B), *M6* mutants exhibited very poor behavioral performance in a simple paradigm. These results suggest that M6 might play a role in higher-order visual processing, because structural alterations do not account for the defective light response. Indeed, immunofluorescence analysis of chaoptin localization (a well characterized marker of fly photoreceptors) in *M6* mutant retinas did not exhibit any clear defect (data not shown). However, this observation does not preclude a role for M6 in retinal morphogenesis. In fact, directing *M6*-RNAi expression to the eye (employing ey-GAL4 and GMR-GAL4) resulted in clear structural defects (data not shown). These results suggest a functional role for M6 in the establishment of the neural circuitry underlying visual processing.

The compound eye develops from a single-layered epithelium, the eye imaginal disc. Pupal eye development involves a coordinated series of morphogenetic events, such as cell-cell communication, differential cell adhesion, maintenance of cell polarity, cell shape, local cell movement, and programmed cell death, to properly pattern the ommatidia in the adult eye [40,41]. Accordingly, we previously reported a role for M6 in maintenance of the follicular epithelia, likely *via* cell adhesion, during cell remodeling [20], which was further supported by the observation of abnormal DE-cadherin distribution in the follicular epithelia of late egg chambers in *M6* hypomorphic mutants (MPZ and MFC, unpublished data). Beta-integrin (*mys*) and M6 genetically interacted, which was supported by fly lethality when *M6*^*03*^ and *mys*^1^ alleles were combined (MPZ and MFC, unpublished data). Recently, our group demonstrated M6a localization in membrane microdomains, which are compatible with lipid rafts in primary hippocampal neuronal cultures [42]. Interestingly, misexpression of the Reggie/Flotilin lipid raft markers in the fly eye imaginal disc results in severe disturbance of the ommatidial pattern and specific and severe mislocalization of cell adhesion molecules [43]. These results suggest a role for M6 in cell-adhesion during eye development.

According to differential tissue expression of transcripts in flies, each variant might play distinct roles in different tissues. Therefore, M6 might play a dual role in cell remodeling in the visual system (present study) and epithelia [20].

### M6 functions in *Drosophila*

In addition to the requirements of M6 during fly development [20], we demonstrated the role of M6 in adult survival. Because the median life span of hypomorphic *M6*^*01*^ males was slightly reduced, results suggest that M6 could also play a role in the regulation of animal aging.

M6 is expressed in several fly neuropils (Figure3), including the central complex, a region involved in control of fly locomotion [44,45]. In addition, preliminary results showed that overall locomotor activity was slightly, although significantly, reduced (approximately 20 %, data not shown) in adult flies with silenced *M6* expression specifically in the ellipsoid body (a central-complex structure) *via* a specific driver (c232-GAL4, [46]), suggesting that M6 could be required in neural circuits underlying this behavior. Notably, PLP mutant mice exhibit deficits in locomotor activity [47]. Our results also showed M6 expression in mushroom bodies. The mushroom bodies are analogous to the mammalian hippocampus, where M6a is abundantly expressed and regulated by chronic stress [3]. In flies, the mushroom bodies are crucial for olfactory learning and memory. Therefore, future studies should evaluate the role of M6 in this complex behavior.

Similarly, it has been reported that M6a expression in the adult brain is stronger in non-myelinated axonal fibers compared with myelinated axons [7]. Because proteolipid genes appeared earlier in evolution than myelin, it has been hypothesized that their involvement in myelination was acquired later [4,5]. Consistent with this, the present study did not detect myelinated axons in *Drosophila*. In flies, nerve ensheathment depends on axonal insulation by glial cells, as well as the subsequent establishment of septate junctions between glial cell membranes. It is worth mentioning that cell junction organization and function share common features in vertebrates and invertebrates [48]. Therefore, M6a/M6 could potentially act as a mediator of cell-cell interactions involved in axon fasciculation during development. This possibility is also supported by the observation that in the *Drosophila* embryo, M6 co-localizes with Fasciclin II, a marker of longitudinal axon fascicles in the nervous system [20].

## Conclusions

In conclusion, we have revealed tissue-differential expression of novel M6 isoforms, one of which was non-functional. *M6* was shown to be the functional fly ortholog of *Gpm6a*. In addition, a role for M6 in the regulation of life span and in the *in vivo* fly visual system was revealed, which is particularly relevant owing to conservation of this protein between flies and mammals.

## Abbreviations

CNS, Central nervous system; Cys, Cysteine; EC, Extracellular; ESEM, Environmental scanning electron microscope; GFP, Green fluorescent protein; Gpm6a, Glycoprotein M6a; IC, Intracellular; NMD, Nonsense-mediated mRNA decay; PCR, Product chain reaction; PLP, Proteolipid protein; qPCR, Quantitative PCR; TM, Transmembrane; UTR, Untranslated region.

## Competing interests

The authors of this manuscript do not have competing financial or non-financial interests in relation to this work.

## Authors’ contributions

MPZ conceived, designed, carried out the experiments, analyzed the data, performed the statistical analysis, and wrote the paper. SCB carried out the molecular and cellular studies. GB carried out immunohistochemistry on adult retinas. ACF conceived the study, analyzed the results, and helped to draft the manuscript. MFC conceived the fly’s experiments, participated in their design, analyzed the results, and helped to draft the manuscript. MAB coordinated the study, helped to design the molecular and cellular experiments, analyzed the results and wrote the paper. All authors read and approved the final manuscript.
